# Borehole Radar Experiment in a 7500 m Deep Well

**DOI:** 10.3390/s25102991

**Published:** 2025-05-09

**Authors:** Huanyu Yang, Kaihua Wang, Yajie Liu, Cheng Guo, Qing Zhao

**Affiliations:** School of Resources and Environment, University of Electronic Science and Technology of China, Chengdu 611731, China; 202321070101@std.uestc.edu.cn (H.Y.); 202212070402@std.uestc.edu.cn (K.W.); liuyajie0719@163.com (Y.L.); guocheng@uestc.edu.cn (C.G.)

**Keywords:** borehole radar, ultra-deep well detection, electromagnetic wave reflection, hydrocarbon exploration

## Abstract

This paper presents the world’s first radar detection experiment conducted in a 7500-m ultra-deep well. By applying ground-penetrating radar technology to petroleum logging, the developed borehole radar system successfully achieved stratigraphic information detection in the 7200–7500 m section of Shunbei Well No. 2. Utilizing electromagnetic wave reflection principles, the system acquires echo signals carrying medium characteristics through transmit–receive antenna arrays coupled with field-programmable gate array (FPGA)-based high-speed acquisition for real-time downhole data transmission. Experimental results demonstrate high consistency in Gamma Ray (GR) curves (correlation coefficient: 0.92) between radar data and Sinopec’s geological drilling data, particularly in key stratigraphic features such as casing reflections at a 7250-m depth (error of 0.013%). This breakthrough validates the operational stability and detection accuracy of borehole radar in complex subsurface environments, providing an innovative technological approach for ultra-deep hydrocarbon exploration.

## 1. Introduction

Underground oil exploration is an important technology in the field of modern energy exploration, which is widely used in the discovery and exploitation of oil resources. With the continuous growth of global energy demand, traditional oil and gas resources tend to be depleted gradually, and new detection technology provides the possibility of finding new oil reserves. Underground oil exploration is a comprehensive process that employs multidisciplinary approaches, including physical, chemical, and geological methods, to investigate the distribution patterns of oil and gas resources at depths ranging from thousands of meters to even greater subterranean levels. This sophisticated exploration process integrates advanced geophysical instrumentation and techniques, such as acoustic wave detection, electrical resistivity tomography, borehole radar imaging, and spontaneous potential measurement, to obtain precise subsurface data and facilitate accurate resource assessment. In recent years, with the rapid development of computer technology, data analysis methods, and materials technology [1,2,3,4], the accuracy and efficiency of underground oil exploration have also been significantly improved. In particular, the combination of geophysical technology and remote sensing technology makes underground oil exploration more refined and intelligent, effectively improving the success rate of resource exploration and the economic benefits of exploitation.

Currently, there are various ways of detecting data in wells. Sonic logging technology is an important technical tool in the field of well logging. It utilizes emitted sound waves and receives reflected, refracted, and transmitted signals in a formation to obtain key information, such as acoustic velocity, attenuation characteristics, and the fracture development of the formation rocks. However, [5] traditional acoustic logging has a low resolution, making it difficult to accurately identify thin layers or subtle formation changes. Natural potential logging is a logging method that measures the changes in naturally occurring potentials in the shaft of a well in a bare borehole to study the stratigraphic properties of the borehole profile [6]. Natural potential logging was one of the first logging methods used in the world. In 1934, C. and M. Schlumberger and E.G. Leonardon published the first paper on electric well logging. The self-potential curve was attributed mainly to the flow potential, i.e., the electrodynamic effect [7]. Resistivity logging is an oil and gas exploration logging technique. It calculates resistivity by emitting or inducing a current downhole into a formation and measuring the formation’s response to that current or electromagnetic field.

Ground-penetrating radar (GPR) operates based on electromagnetic property variations between subsurface media. Differences in dielectric constants and conductivities induce electromagnetic wave reflections at lithological interfaces, making GPR an effective geophysical method [8]. Electrical conductivity serves as the primary determinant of electromagnetic wave attenuation, with higher energy losses correlating with stronger conductive properties. Relative permittivity predominantly influences direct wave travel time [9]. The system transmits electromagnetic pulses into geological formations via a transmitting antenna, while a receiving antenna captures reflected waveforms. Quantitative analysis of amplitude and phase characteristics of these echoes enables precise determination of medium parameters. This operational mechanism has established GPR’s widespread application in coal mining exploration, petroleum reservoir characterization, and civil engineering investigations [10,11,12].

Borehole radar (BHR), a specialized adaptation of ground-penetrating radar technology, enables formation characterization at 100–3000 m radial depths in wellbores through electromagnetic interrogation. This advanced logging modality demonstrates a superior spatial resolution (>0.5 m) and an enhanced penetration range compared to resistivity-based logging systems [13]. Quantitative analysis of these reflected waveforms permits non-invasive determination of subsurface features, including fluid contacts and stratigraphic discontinuities. This capability has established BHR’s critical role in modern reservoir evaluation workflows.

In order to obtain deeper stratigraphic information and to meet data quality requirements [14], borehole radar systems have been improved from generation to generation. And deep-well cables are thousands of meters long, making the available bandwidth narrow. Generally, the length of these logging cables is more than 5000 m, but they can be more than 10,000 m long in some cases [15]. This affects the communication rate and quality and limits the data transmission rate. In addition, the narrow space inside a well also limits the space for hardware circuits [16,17]. And radar built-in instruments work in the harsh high-temperature and high-pressure environment of the well. In order to work properly in this harsh environment, not only does the instrument casing need an extremely high tensile strength, but the hardware circuitry also needs to be temperature- and pressure-resistant. This further increases the difficulty of hardware circuit design and increases the difficulty of data transmission. All of these factors have constrained breakthroughs in borehole radar.

Therefore, in view of the shortcomings and limitations of the above logging system, the borehole radar system was selected for the exploration of the Shunbei No. 2 well. And the maximum depth is 7.5 km.

## 2. Principles of Radar in Wells

### 2.1. Principles of Borehole Radar Antennas

**Figure 1:**
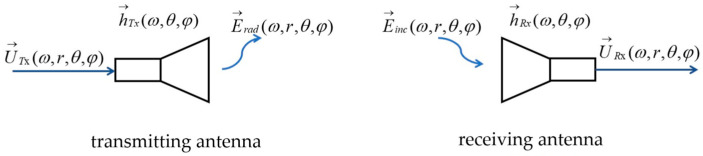
Transmission characteristics modeling diagram for transceiver antenna.

First of all, one of the most important characteristics of a time-domain antenna is to transmit or receive as many carrier-free pulse signals as possible without distortion. Thus, the index used to measure the distortion of the antenna transmitting and receiving signals is particularly important. In order to facilitate the analysis, we established a transceiver antenna transmission characteristics model; the model is shown in Figure 1.

In the model of Figure 1, the feed voltage of the transmitting antenna is ${\overset{\to }{U}}_{Tx}(\omega ,r,\theta ,\varphi )$, the transmission function of the transmitting antenna is ${\overset{\to }{h}}_{Tx}(\omega ,\theta ,\varphi )$, the transmission function of the receiving antenna is ${\overset{\to }{h}}_{Rx}(\omega ,\theta ,\varphi )$, the received pulse voltage of the receiving antenna is ${\overset{\to }{U}}_{Rx}(\omega ,r,\theta ,\varphi )$, the electric field radiated by the transmitting antenna is ${\overset{\to }{E}}_{rad}(\omega ,r,\theta ,\varphi )$, and the incident electric field of the receiving antenna is ${\overset{\to }{E}}_{inc}(\omega ,r,\theta ,\varphi )$. Therefore, the feed voltage of the transmitting antenna and the radiated electric field of the transmitting antenna and the received pulse voltage of the receiving antenna and the incident electric field of the receiving antenna satisfy the relationships of Equations (1) and (2).

(1) $$\frac{{\overset{\to }{E}}_{rad}(\omega ,r,\theta ,\varphi )}{\sqrt{z_{0}}}={\overset{\to }{h}}_{Tx}(\omega ,\theta ,\varphi )\frac{e^{-j\omega \frac{r}{c}}}{r}\frac{{\overset{\to }{U}}_{Tx}(\omega ,r,\theta ,\varphi )}{\sqrt{z_{c}}}$$

(2) $$\frac{{\overset{\to }{U}}_{Rx}(\omega ,r,\theta ,\varphi )}{\sqrt{z_{c}}}={\overset{\to }{h}}_{Rx}(\omega ,\theta ,\varphi )\frac{{\overset{\to }{E}}_{inc}(\omega ,r,\theta ,\varphi )}{\sqrt{z_{0}}}$$

where *z_c_* is the antenna port characteristic impedance and *z*_0_ is the characteristic impedance in free space. When the radiated electric field of the transmitting antenna and the incident electric field of the receiving antenna are taken from the same location, the two are the same electric field, and at this time they satisfy the following equation:

(3) $${\overset{\to }{E}}_{rad}(\omega ,r,\theta ,\varphi )={\overset{\to }{E}}_{inc}(\omega ,r,\theta ,\varphi )$$

Thus, the relationship between the feed voltage of the transmitting antenna and the received voltage of the receiving antenna can be obtained from Equations (1)–(3):

(4) $$\frac{{\overset{\to }{U}}_{Rx}(\omega ,r,\theta ,\varphi )}{{\overset{\to }{U}}_{Tx}(\omega ,r,\theta ,\varphi )}={\overset{\to }{h}}_{Tx}(\omega ,\theta ,\varphi ){\overset{\to }{h}}_{Rx}(\omega ,\theta ,\varphi )e^{-j\omega \frac{r}{c}}$$

Fixing the positions of the two antennas, *r*, *θ*, and *φ* are constants, at which point Equation (4) can be simplified to the following:

(5) $$\frac{{\overset{\to }{U}}_{Rx}(\omega )}{{\overset{\to }{U}}_{Tx}(\omega )}={\overset{\to }{h}}_{Tx}(\omega ){\overset{\to }{h}}_{Rx}(\omega )e^{-j\omega \frac{r}{c}}$$

In general, the antenna’s transfer function is a complex function, so the transfer function of the transceiver antenna can be written in exponential form:

(6) $${\overset{\to }{h}}_{Tx}(\omega )=\left|h_{Tx}(\omega )\right|e^{j\phi_{Tx}(\omega )}$$

(7) $${\overset{\to }{h}}_{Rx}(\omega )=\left|h_{Rx}(\omega )\right|e^{j\phi_{Rx}(\omega )}$$

Thus, Equation (5) can then be written as follows:

(8) $${\overset{\to }{U}}_{Rx}(\omega )=\left|h_{Tx}(\omega )\right|\left|h_{Tx}(\omega )\right|{\overset{\to }{U}}_{Tx}(\omega )e^{-j(\omega \frac{r}{c}-\phi_{Tx}(\omega )-\phi_{Rx}(\omega ))}$$

Assuming Equation (8), the following conditions are satisfied:

(9) $$\left\{\begin{array}{l} \;\left|h_{Tx}(\omega )\right|=a\\ \;\left|h_{Rx}(\omega )\right|=b\\ \;\phi_{Tx}(\omega )=k_{1}\omega \\ \;\phi_{Rx}(\omega )=k_{2}\omega \end{array}\right.$$

where *a*, *b*, *k*_1_, and *k*_2_ are constants. Therefore, according to the displacement theorem of the Fourier transform, the Fourier inverse transform of Equation (8) is performed to obtain the time-domain waveform relationship of the transceiver signal:

(10) $$u_{Rx}(t)=a\times b\times u_{Tx}[t-(\frac{r}{c}-k_{1}-k_{2})]$$

At this point, it can be seen from Equation (10) that the received signal is delayed in time from the transmitted signal, and its amplitude is a×b times that of the transmitted signal. Thus, it can be concluded that if the characteristics of the transceiver antenna satisfy Equation (9), then the transmission of the signal between the receiving antenna will not be distorted. The specific conditions for translating Equation (9) to an antenna are that the antenna has an amplitude–frequency characteristic curve with constant amplitude values in the band covering the signal bandwidth and that the antenna has linear phase-frequency characteristics.

#### 2.1.1. Antenna Characterization in a Well Environment

Dipoles are widely used in the design of radars in wells due to their elongated form factor. The surface current distribution, *I*(*z*), and the input impedance characteristic, *Z_in_*, of the dipole, which are the key parameters of the antenna, have been fully investigated in the well-hole environment. And researchers have derived the relevant mathematical expressions [18].

(11) $$I(z)=\frac{jV_{0}}{2\zeta_{1}\Psi }\frac{\sin[k_{L}(l-\left|z\right|)]}{\cos(k_{L}l)}$$

(12) $$Z_{in}=-2jZ_{c}\cot(k_{L}l)$$

(13) $$k_{L}=k_{1}{\left\{\frac{k_{2}^{2}\left[H_{0}^{\left(2\right)}(k_{2}b)+k_{2}b\ln(b/a)H_{1}^{\left(2\right)}(k_{2}b)\right]}{k_{1}^{2}H_{0}^{\left(2\right)}(k_{2}b)+k_{2}^{3}b\ln(b/a)H_{1}^{\left(2\right)}(k_{2}b)}\right\}}^{1/2}$$

(14) $$\left\{\begin{array}{c} \zeta_{1}={\left[\mu_{0}/(\varepsilon_{2}-j\frac{\sigma_{2}}{\omega })\right]}^{1/2}\\ k_{1}=\omega {\left[\mu_{0}/(\varepsilon_{2}-j\frac{\sigma_{2}}{\omega })\right]}^{1/2}\\ k_{2}=\omega {\left[\mu_{0}/(\varepsilon_{4}-j\frac{\sigma_{4}}{\omega })\right]}^{1/2} \end{array}\right.$$

(15) $$\Psi =\frac{k_{L}}{2\pi k_{1}}\left[\ln(b/a)+\frac{k_{1}^{2}}{k_{2}^{2}}\frac{H_{0}^{\left(2\right)}(k_{2}b)}{k_{2}bH_{1}^{2}(k_{2}b)}\right]$$

(16) $$Z_{c}=\zeta_{1}\Psi =\frac{\zeta_{1}k_{L}}{2\pi k_{1}}\left[\ln(b/a)+\frac{k_{1}^{2}}{k_{2}^{2}}\frac{H_{0}^{\left(2\right)}(k_{2}b)}{k_{2}bH_{1}^{2}(k_{2}b)}\right]$$

Here is a Hankel function of the second class of nth order, and the conditions under which Equation (11) is valid are as follows:

(17) $$\left\{\begin{array}{c} \left|\frac{k_{4}^{2}}{k_{2}^{2}}\right|\geq 2\\ k_{2}b<<1 \end{array}\right.$$

As shown in the surface current, the input impedance expression of the dipole antenna in the well-bore environment and the performance characteristics of the antenna are related to its own structural properties. They are also related to the size of the well bore, the well-bore filling substance, and the well wall material.

#### 2.1.2. Antenna Bandwidth Enhancement Mechanism

**Figure 2:**
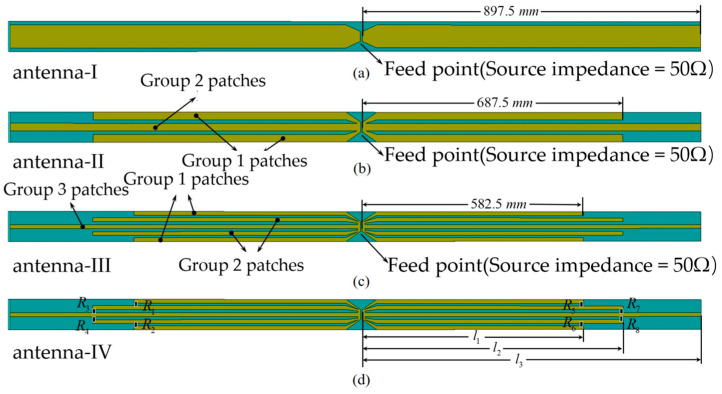
Structure of the four antennas: (**a**) antenna-I; (**b**) antenna-II; (**c**) antenna-III; (**d**) antenna-IV.

The patch located at the right end of the microstrip sheet is an arm of an asymmetric omnidirectional dipole. It consists of three sets of metal sheets of different lengths to broaden the bandwidth of the dipole. As shown in Figure 2, in order to analyze the mechanism of bandwidth broadening of the asymmetric omnidirectional dipole antenna, in this paper, antenna-I, antenna-II, antenna-III, and antenna-IV were designed. Antennas-I to -III are dipole antennas consisting of one, two, and three sets of metal patches of different lengths, respectively.

**Figure 3:**
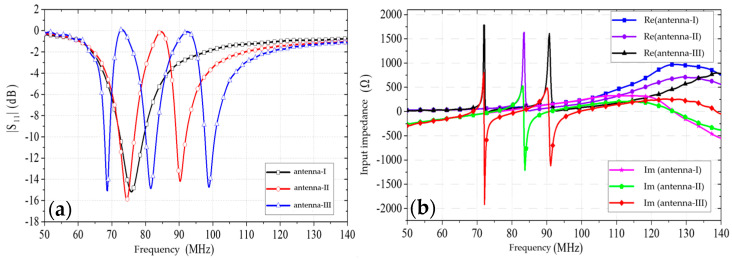
Simulation results for antennas I–III: (**a**) |S11| values of the antennas; (**b**) input impedance values of the antennas.

The designed antennas were simulated using CST Studio Suite 2022 simulation software. The reflection coefficients (|S11|) and input impedances of antennas-I to -III are shown in Figure 3. From the reflection coefficients (|S11|) of the three antennas, it can be seen that the number of resonance points of the antennas is equal to the number of groups of the antennas with different lengths of patches. However, there is a great reflection in the band located between the two resonance points. This makes it impossible to utilize multiple resonance point reconstruction and thus broaden the bandwidth of the antennas. As shown in Figure 3b, RE is an acronym for the real part and IM is an acronym for the imaginary part. The input impedance of the antenna in the band between the two resonance points is very high, which leads to strong reflections in the band between the resonance points.

From the above results, it is clear that there is mutual interference between patches of different lengths. This leads to excessive input impedance between the resonance points generated by patches of different lengths, which in turn results in port mismatch. Therefore, it is not feasible to simply use dipole antennas of different lengths for frequency reconstruction to achieve antenna bandwidth broadening. From the results on the input impedance of the antennas, if the reconfiguration of the antenna frequency is to be realized, the input impedance of the antenna between the resonant frequency points must be reduced.

**Figure 4:**
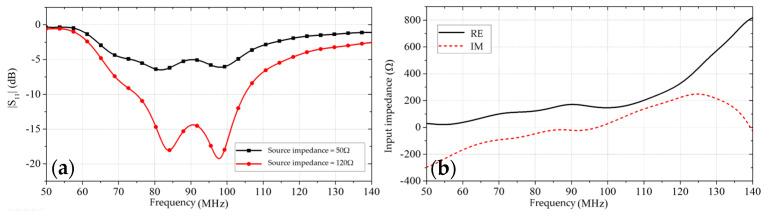
|S11| and input impedance of antenna-IV: (**a**) |S11|; (**b**) input impedance.

Therefore, in this paper, antenna-IV was designed. As shown in Figure 2d, antenna-IV still uses three sets of patches of different lengths to combine into a dipole. The lengths of the three sets of patches are *l*_1_ = 582.5 mm, *l*_2_ = 687.5 mm, and *l*_3_ = 897.5 mm, respectively. And resistors are connected across the patches of different lengths to reduce the input impedance of the antenna between resonant frequency points. The values of the loaded resistors are *R*_1_ = 1.5 kΩ, *R*_2_ = 1.5 kΩ, *R*_3_ = 1.5 kΩ, *R*_4_ = 1.5 kΩ, *R*_5_ = 1.5 kΩ, *R*_6_ = 1.5 kΩ, *R*_7_ = 1.5 kΩ, and *R*_8_ = 1.5 kΩ. The |S11| of antenna-IV as well as the input impedance are shown in Figure 4. From the figure, it can be seen that the impedance of antenna-IV, after spanning the resistor, does not show jump change between resonant frequency points. The input impedance of the antenna remains basically the same in the operating frequency range, and the frequency reconstruction of the antenna is completed.

### 2.2. Principles of Radar Emission

#### 2.2.1. Principles of Pulse Signals

Shock pulse signals are currently the most widely used method for ground-penetrating radar transmitting systems. Generally, conventional shock pulse signals are similar to Gaussian pulse signals with an extremely narrow pulse width in the time domain. According to the Fourier transform, a signal is extremely narrow in the time domain and extremely wide in the frequency domain. Since there is an extremely wide frequency domain, it responds well to complex subsurface information [19], and the time-domain expression [20] for a Gaussian pulse is as follows:

(18) $$f\left(t\right)=\frac{1}{\sqrt{{2\pi \sigma }^{2}}}e^{\left(-\frac{t^{2}}{{2\sigma }^{2}}\right)}$$

This is obtained by making *σ*^2^ = α^2^/4*π*:

(19) $$f(t)=f\left(t\right)=\pm \frac{\sqrt{2}}{\alpha }e^{\left(-\frac{2\pi t^{2}}{\alpha^{2}}\right)}=\pm A_{p}e^{\left(-\frac{2\pi t^{2}}{\alpha^{2}}\right)}$$

**Figure 5:**
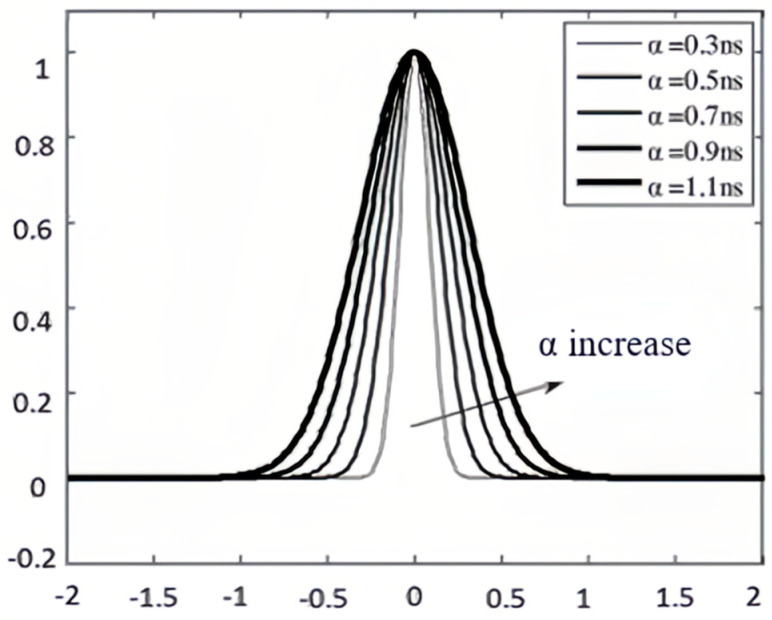
The time-domain waveforms of Gaussian pulses corresponding to different α values.

As shown through Equation (19), when α decreases, the magnitude of f(t) becomes larger. At the same time, since α is the denominator of the negative power, the smaller α is, the faster the power function decays, so it will lead to a smaller width of the whole pulse. A directly affects the amplitude of the pulse signal. Since there is a direct relationship between energy and amplitude, it can directly reflect the energy of the pulse. Generally, α is called the shaping factor. Figure 5 shows the time-domain waveform of a Gaussian pulse when the shaping factor, α, takes the values of 0.3 ns, 0.5 ns, 0.7 ns, 0.9 ns, and 1.1 ns. The pulse amplitude has been normalized.

**Figure 6:**
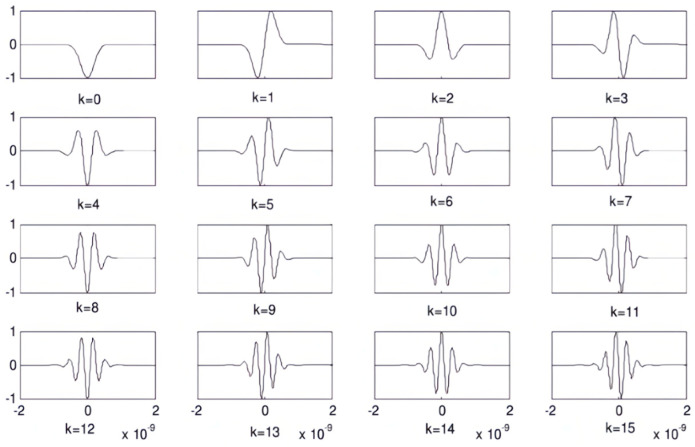
The time-domain waveforms of the 0th-to 15th-order derivatives of the Gaussian pulse.

According to the formula of the Gaussian pulse time-domain waveform, a Gaussian pulse signal of nanosecond order is generally obtained by adjusting the magnitude of the pulse shaping factor, α. The Gaussian pulse is calculated by derivation to obtain a Gaussian pulse signal of a higher order. The higher the derivation order, K, the more pulse peaks the Gaussian pulse signal will produce. Thus, it will make the main peaks insignificant. The inconspicuousness of the main peak of the pulse will have an adverse effect on the subsequent acquisition system to recognize the pulse peak and process the echo data. So, extracting the main peak signal will not only increase the design difficulty of the acquisition system but also increase the BER of the system. Due to the complexity of the Gaussian pulse waveform generated after multiple differentiation, this approach is unfavorable. At the same time, a high-order Gaussian pulse will increase the design difficulty of the transmitter circuit, which is unfavorable to the realization of the transmitter source circuit. It is generally believed that the smaller the order of the Gaussian pulse, the more easily its waveform can be realized. The specific 0–15 order of each Gaussian pulse order is shown in Figure 6.

**Figure 7:**
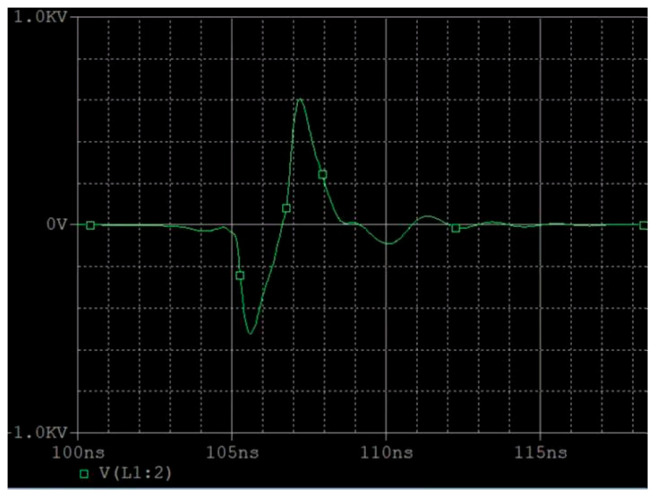
First-order Gaussian pulse time-domain waveform.

The first-order Gaussian pulse signal simulation is shown in Figure 7. Through Figure 7, it can be seen that the Gaussian pulse signal is only of nanosecond order. The center frequency of this pulse signal is higher, so the sampling rate of the acquisition system also has higher requirements. The specific relationship between the current pulse radar and the detection depth is shown in Table 1. From the table, it can be seen that there is a clear point of contradiction between the detection depth and the depth resolution. The depth resolution of the system decreases significantly when the probing depth is deeper. So, in order to cope with different needs, the center frequency of the ground-penetrating radar system will also be specially designed. Therefore, the ground-penetrating radar acquisition system also has higher requirements. Table 1 shows the parameters under the assumed conditions. The loss of electromagnetic waves by the propagation medium at the time of detection is less than 20 dB/m. The relative permittivity of this propagation medium is εr = 9. The pulse width of the radar receiver is calculated based on the half-power point of the signal.

#### 2.2.2. Pulse Signal Generation

**Figure 8:**
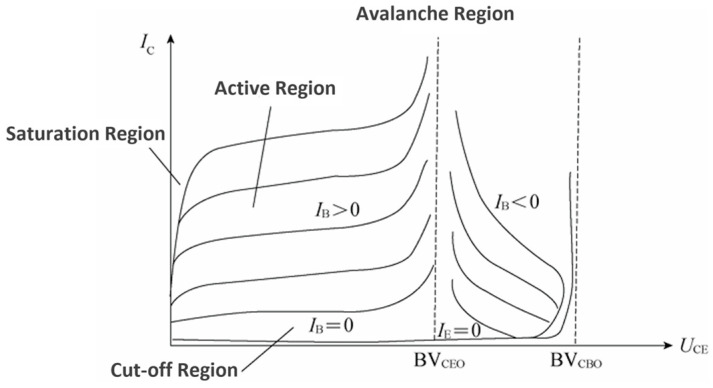
Avalanche tube output characteristic curve.

The avalanche transistor was developed by S. L. Miller and J. J. Ebers of Bell Laboratories in 1955 [21]. Figure 8 shows the output characteristic curve of an avalanche transistor, which is often used to generate pulse generation signals for radar detection because of its avalanche multiplication effect.

**Figure 9:**
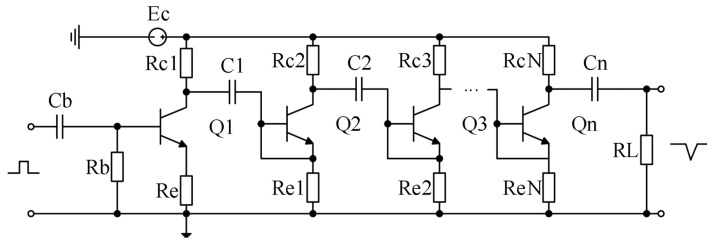
Single-load Marx circuit.

According to the circuit of the Marx generator, it is improved to obtain the Marx circuit suitable for single-load radar in wells (Figure 9). The basic principle is parallel charging and series discharging. The Q1 tube is responsible for the triggering conduction, and the Q2–Qn tubes are sequentially responsible for the over-voltage conduction, which generates a negative pulse on the load, RL.

**Figure 10:**
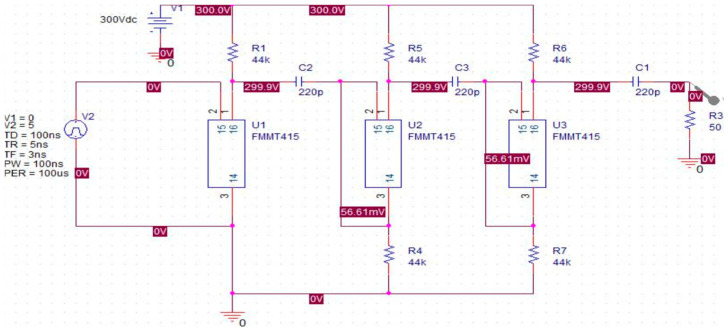
Three-level Marx simulation diagram.

The following is a simulation of the Marx circuit through the spice model of the FMMT415 avalanche tube from Zetex (Oldham, UK). Taking the three-stage Marx circuit as an example, a simulation schematic is shown in Figure 10. The charging voltage is 300 V, the charging capacitor is 220 pF, and the isolation and charging resistors are 44 k ohms. The trigger pulse is a rectangular narrow pulse with a trigger frequency of 10 kHz and a load resistance of 50 ohms.

**Figure 11:**
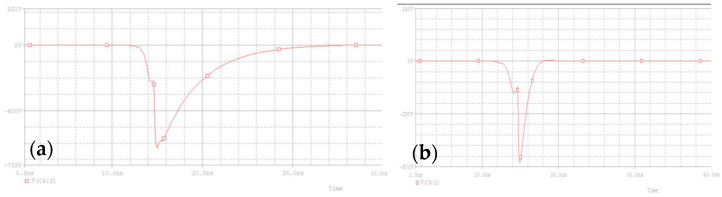
Simulation output waveform: (**a**) without inductance; (**b**) with sharpened capacitors and inductance added.

By placing a voltage probe on the load resistor, the observed voltage waveform is shown in Figure 11a. It can be seen that the negative pulse generated has a narrow pulse front and a wide pulse back delay. The leading edge is mainly determined by the conduction speed of the avalanche tube, which is generally around 2 ns. It is seen as a Gaussian waveform. The trailing edge due to the discharge loop Resistor Capacitance (RC) mainly shows an exponential decay curve. In order to make the waveform closer to the Gaussian pulse, generally, the last level of sharpening capacitance, that is, a small capacitor, is used to allow the discharge process to cut off faster. At the same time, an inductor is added after the capacitor, generally at the level of nH, to slow down the rate of rise of the leading edge. The output waveform is shown in Figure 11b.

### 2.3. Radio Frequency (RF) Acquisition Principle

The principle behind the design of the acquisition system is the Nyquist Sampling Theorem. That is, when sampling a signal, the sampled signal frequency must be greater than twice the highest frequency of the target signal. In order to better recover the original signal, at least 5–10 times the highest frequency of the original signal must be achieved. We used the real-time sampling technique for RF signal acquisition.

**Figure 12:**
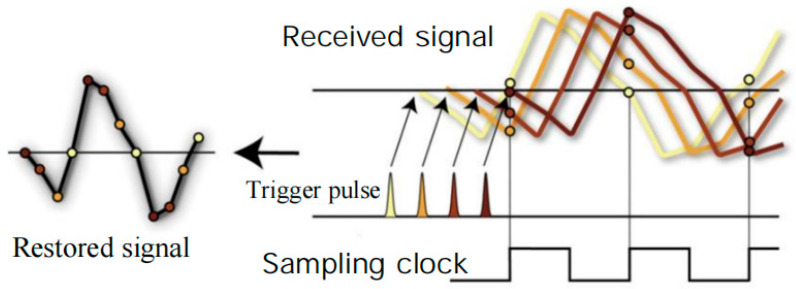
Time-division sampling.

Real-time sampling techniques use clock intervals to sample the target signal. A large number of waveform points can be obtained in a single sampling. The reconstruction of the echo waveform is performed based on the sampling points obtained. The real-time sampling technique can be categorized as a single analog-to-digital converter (ADC), multichannel ADC, or multichip ADC depending on the number of ADCs used, where the multichannel ADC or multichip ADC uses the time-alternating sampling technique. By shifting the clock signal, a phase shift operation is performed on the clock signal according to the number of ADCs. The control of multiple ADCs is accomplished in one time cycle, and then the waveform is recovered according to the time sequence. The specific schematic is shown in Figure 12.

According to the definition of the alternating-time sampling technique, it can be seen that this technique does not have high ADC requirements. There is no need to use an ADC with a very high sampling rate, considering the large size of the board for the alternating-time sampling technique. There is also the effect of clock shifting on the ADC. So, it was decided not to use this approach in this design.

**Figure 13:**
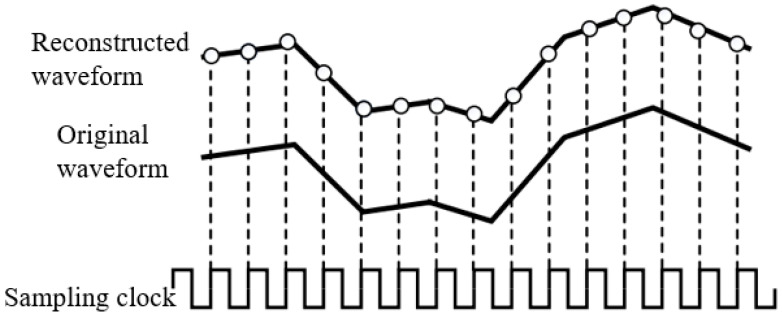
Real-time sampling.

In the real-time sampling technique using a single ADC, an ADC is used to sample enough points in a time cycle to complete the reconstruction of the waveform. This method is good for sampling both periodic and non-periodic signals. The specific sampling schematic is shown in Figure 13. But due to the Nyquist sampling theorem mentioned above, the sampling rate of monolithic ADCs should be 5–10 times the highest frequency of the target signal. So, this method requires a high sampling rate and resolution for the ADC. With the introduction of the JESD204B high-speed serial protocol, ADCs from various manufacturers can now use the JESD204B protocol to achieve a Giga Samples Per Second (GSPS) sampling rate. Considering the method of use, it is necessary to complete the selection of ADC and FPGA according to the project requirements.

### 2.4. Well-Ground Communication Principles

**Figure 14:**
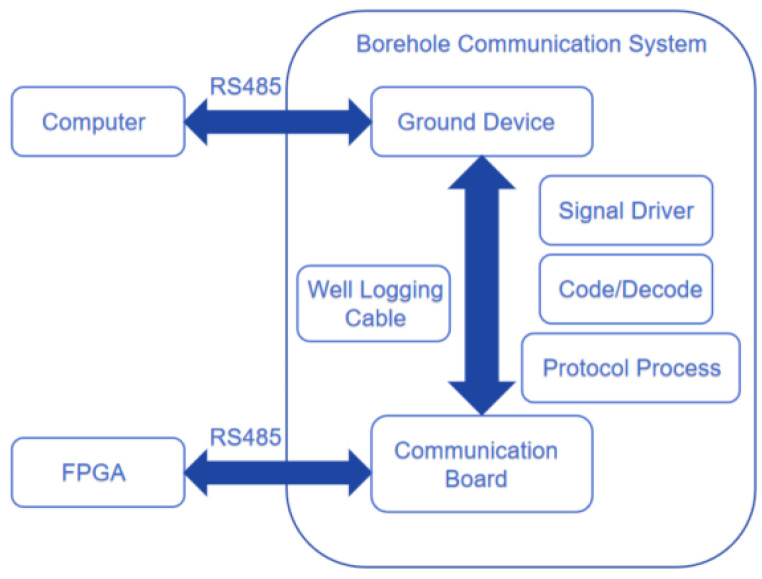
Well logging telemetry system.

Figure 14 is a block diagram of the structure of the radar system in the well. It is mainly composed of the surface system and the downhole radar system. These two parts communicate with each other through cables. The downhole FPGA transmits the radar data to the surface system through the cable and then processes the data. At the same time, the ground system sends commands to the downhole FPGA to transmit the data.

**Figure 15:**
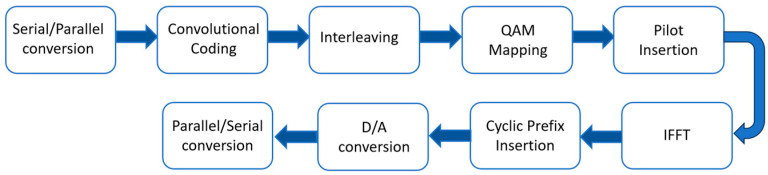
Process of OFDM system transmission.

The communication method uses OFDM (Orthogonal Frequency Division Multiplexing), which is most commonly used for radar in wells for data transmission. The OFDM modulation technique belongs to a special type of multicarrier modulation. High-speed serial data are converted into parallel low-speed data, which are transmitted over different orthogonal subchannels by different subcarriers. OFDM allows the spectral overlap of the subcarriers. The OFDM communication system can be divided into two parts: data transmission and data reception. The general structure of the data sending system is shown in Figure 15.

On the transmitter side, the borehole radar data acquisition system packages the collected data into the borehole radar communication system via an RS485 bus. They pass through scrambling code, convolutional coding, packet interleaving, QAM-level mapping, and insertion of a frequency guide in sequence in the FPGA. After the inverse fast Fourier transform (IFFT), the cyclic prefix is added. It is converted into a differential analog signal by DAC and then sent to the cable through a differential amplifier. The receiving end of the host computer is the inverse of the transmitting end.

#### 2.4.1. Scrambling Code

**Figure 16:**
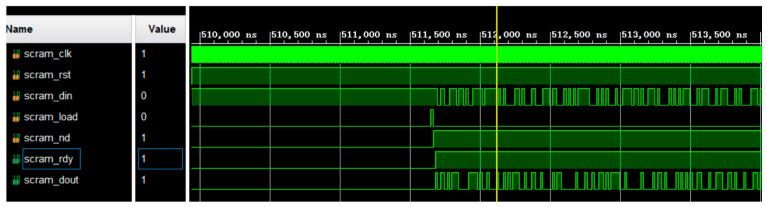
Simulation results for scrambling code.

After a frame of data from the radar acquisition system in the well is received, it has to be stored in the first-in–first-out (FIFO) module first, and then 9 bytes of data at a time are read from the FIFO to feed them into the scrambler module. Figure 16 shows the simulation diagram. Driven by the clock, after the scrambler enable signal, SC_EN, is pulled high from a low level, the scrambler loads the initial value in the register. The operation proceeds according to the scrambler formula. The result of the operation is an input to the first stage, and the scrambled data and data-valid signal are output after the result is differentiated from the input serial data.

#### 2.4.2. Convolutional Coding

**Figure 17:**
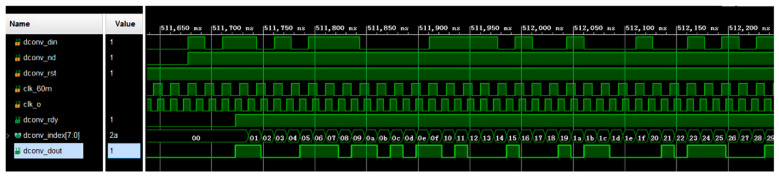
Simulation results for convolutional coding.

Perturbation code processing is followed by grouped convolutional coding. Convolutional code (n, k, and m) is an error correction code with memory. It consists of shift registers, switches, and adders [22]. As shown in Figure 17, when the input signal is valid, the result of convolutional coding is obtained by the different-or operation of the shift register driven by the clock. One bit is encoded to obtain two bits with increased redundancy. In order to increase the efficiency of data transmission, regular deletion of redundancy is required.

#### 2.4.3. Interleaving

After scrambling and convolutional coding, the technique of group interleaving is required to reduce the burst errors. The data are fed into the interleaver in the order of rows and then output from the interleaver in the order of columns. In this scheme, two dual-port random access memories (RAMs) are used as data cache FIFOs for two-stage interleaving.

**Figure 18:**
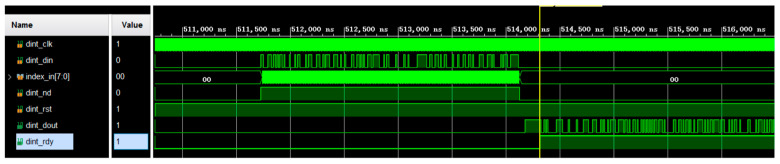
Interleaving simulation results.

As shown in Figure 18, dint_din is the data inputted by the convolutional coding module. index_in [7:0] is the corresponding sequence number of the data. ddin_nd is the data-valid signal. After the data pass through the two levels of the interleaving module, the output interleaved result is finally obtained.

#### 2.4.4. Quadrature Amplitude Modulation (QAM) Mapping

**Figure 19:**
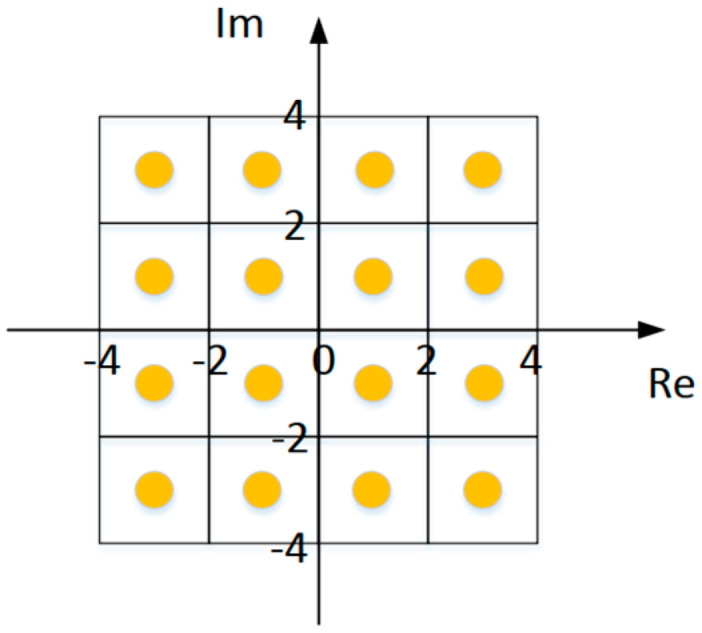
QAM mapping constellation diagram.

QAM modulation is also known as quadrature amplitude modulation [23]. In this design, in order to improve the noise immunity and also to increase the throughput of the system, combining the needs of both, a more balanced scheme of 16-QAM is adopted. The adopted constellation diagram is shown in Figure 19. Sixteen constellation points represent sixteen vector points. The horizontal axis represents the real part, and the vertical axis represents the imaginary part. A vector point consists of four bits of data: two bits for the real part and two bits for the imaginary part. By mapping the data for the real part, 00, 01, 11, 10 to −3, 1, 1, and 3, on the horizontal axis and mapping the data for the imaginary part, 00, 01, 11, 10 to −3, −1, 1, and 3, on the vertical axis, the serial bits output from the packet interleaving can be mapped to the corresponding constellation points.

**Figure 20:**
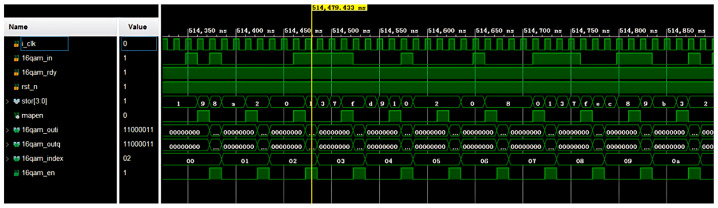
16-QAM mapping simulation results.

As shown in Figure 20, each of the 4 bits of data input first undergoes serial–parallel conversion and is stored in the register stor [3:0]. After full storage, the multiplexer maps the real and imaginary parts of a QAM symbol based on the values of stor [3:2] and stor [1:0], respectively. The real and imaginary parts can map four different levels. That is, 16 vector points can be generated. The final outputs are the two (I and Q) QAM signals 16qam_outi and 16qam_outq. The data-valid signal 16qam_en is pulled high, and the QAM data number 16qam_index is incremented by one until the end of the frame. The counter is reset and waits for the input of the next frame.

#### 2.4.5. Pilot Insertion

**Figure 21:**
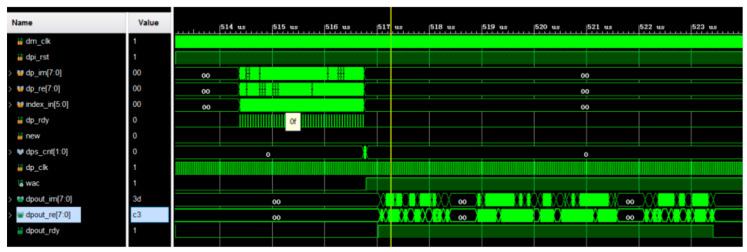
Simulated waveforms of guided-frequency insertion.

The backend module for QAM-level mapping is the lead-in insertion module. At the time of long-distance transmission over the cable, the attenuation and noise in the cable is greater. In order for the receiver to obtain information about the characteristics of the channel, the channel needs to be estimated. The function of the FG (frequency guide) is to transmit a known sequence. By comparing the received FG signal with the known FG signal, the receiver can determine the amplitude and phase deviation of the signal attenuation after passing through the cable and obtain the equalization coefficient. Thus, it compensates for other transmitted data and recovers the original signal. As shown in Figure 21, the real and imaginary signals of the complex signal generated by the previous level of QAM modulation enter the frequency-conducting insertion module. When a frame input is completed, the wac signal is pulled high. The write address is set according to the address index and stored in RAM. When all the input QAM-modulated data are stored in RAM, the new_dp signal is pulled high to start inserting the guide frequency. The read enable is turned on when the insertion of the four leads is completed. Output of the adjusted order of the data starts, and finally a set of constructed complex conjugate sequences with a length of 128 is constructed.

#### 2.4.6. Inverse Fast Fourier Transform (IFFT)

**Figure 22:**
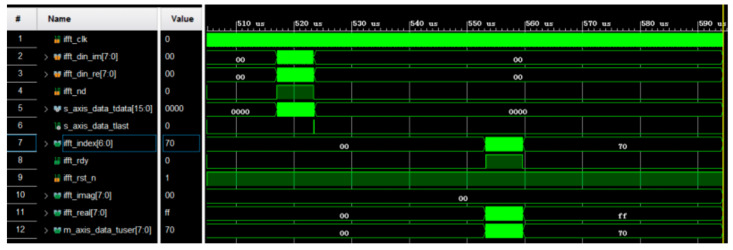
Simulation results of IFFT transform.

**Figure 23:**
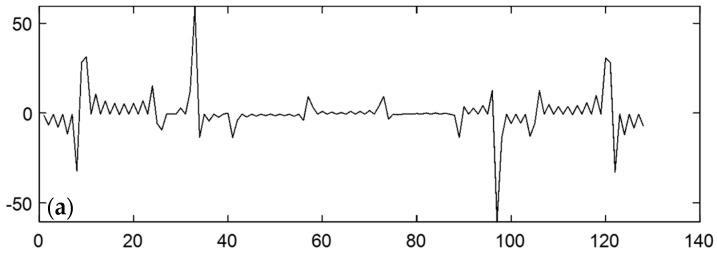

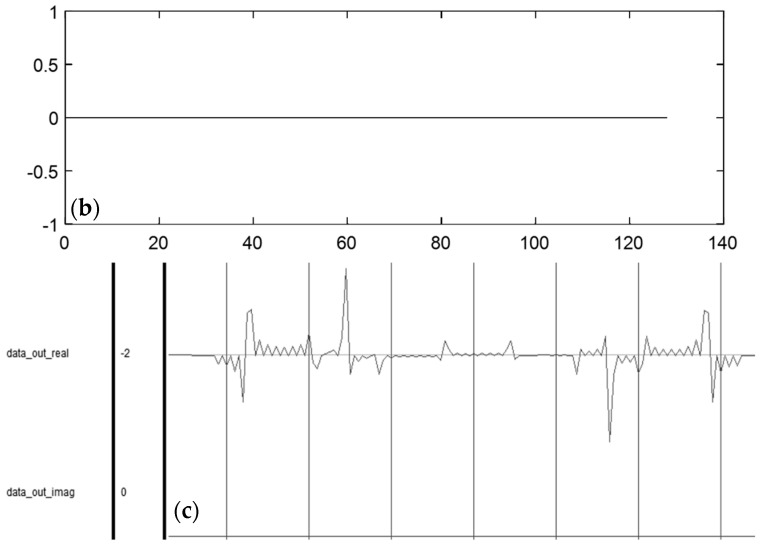
Comparison of IFFT: (**a**) MATLAB simulation real-part results; (**b**) MATLAB simulation imaginary-part results; (**c**) IFFT module of Vivado simulation results.

After going through QAM mapping, the interleaved data are mapped onto real and imaginary signals. These QAM symbols need to undergo an IFFT transform to realize OFDM modulation. The IFFT algorithm used in this design is based on the Fast Fourier Transform IP core integrated in Xilinx Vivado. As shown in Figure 22, after the IP core is configured, the real data inputted by the FFI module go into the lower eight bits of s_axis_data_tdata. The imaginary data go into the high octet of s_axis_data_tdata. The valid data signal is used as input to the s_axis_data_tvalid interface. The final output OFDM signal has an imaginary part of 0. The Matlab R2014a verification result is shown in Figure 23.

Figure 23 shows that the complex conjugate sequence is restored to the original complex conjugate sequence after the IFFT transform in FPGA and then the fast Fourier transform (FFT) in matlab. This proves the correctness of the IFFT transform module.

## 3. Data Collection and Processing

**Figure 24:**
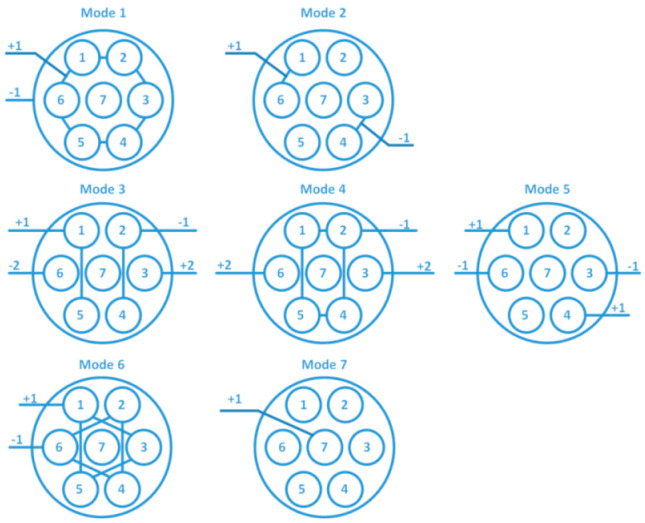
Operating mode of the cable [24].

In order to realize data detection in ultra-deep well environments, we completed a validation comparison of actual 7500 m deep well measurements in the Shunbei area. First of all, there were seven operating modes of the cable, according to Figure 24. Mode I and mode VII were single-ended transmissions with poor signals. Mode III and mode IV had high voltage drive requirements, and mode VI occupied too many cable cores, affecting the power supply. So, we used the mode II connection with a baud rate of 38,400.

**Figure 25:**
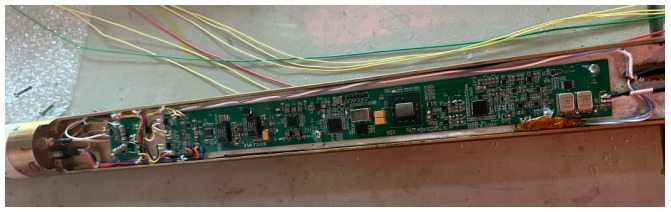
Communication board and FPGA.

The communication board and FPGA acquisition board were placed in an outer protective sleeve, as shown in Figure 25, and then descended from a depth of 7200 to a 7500 depth at 10 m/min.

**Figure 26:**
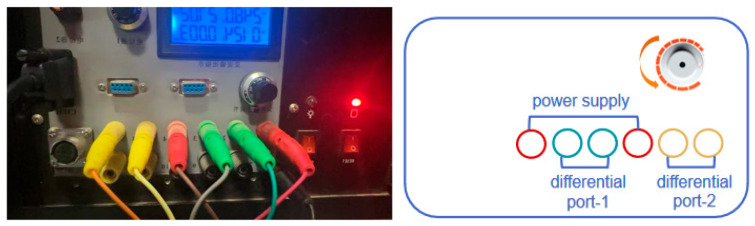
Ground telemetry board.

Figure 26 shows a schematic of the terrestrial system, with a knob above it to adjust the impedance of the terrestrial chassis to match the impedance of the chassis and the cable to minimize the return loss of the cable transmission.

### 3.1. Detection Data Collection in the Shunbei Region

In the Shunbei area, several drilled wells were explored using an improved borehole radar system. Geological data were collected at a depth of 7500 m below ground. To ensure the completeness and accuracy of the data, multiple repetitive measurements and real-time data monitoring were used during the acquisition process.

The time-domain waveform can be used to visually determine whether a signal has significant distortion, abnormal spikes, or noise interference. From the time-domain signal in the above figure, it can be seen that there were periodic changes. This indicates that the radar system successfully captured the main signal components. However, there is still a certain amount of high-frequency noise in the waveform, which may have been caused by ring interference or system noise.

**Figure 27:**
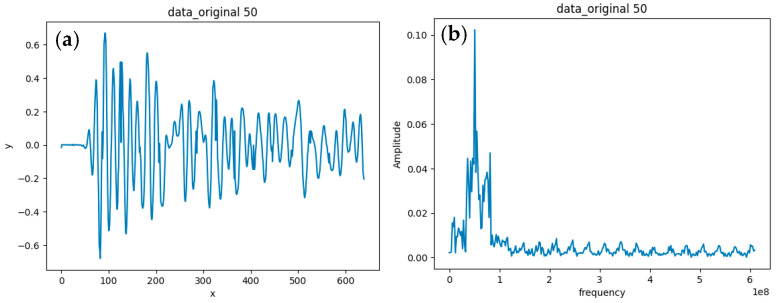
Single-channel time-domain waveform and frequency-domain characteristics: (**a**) time-domain A-scan; (**b**) A-scan in the frequency domain.

Frequency-domain signals are converted from time-domain signals into spectral distributions by Fourier transform to show the amplitude distribution characteristics of the signal for each frequency component. The frequency-domain analysis not only helps to detect the frequency response and operating bandwidth of the radar system, it also effectively identifies the distribution of noise. It can be observed from the Figure 27 that the main frequency components in the low-frequency region are more significant. This indicates that the signal energy is mainly concentrated in the low-frequency range, while the amplitude of the high-frequency region is smaller, indicating that the high-frequency noise is well suppressed.

In addition, the operating status of the system can be further verified by analyzing the center frequency of the received waveform with the center frequency of the known antenna design. This analysis shows that the center frequency of the received waveform is consistent with the center frequency of the antenna. This indicates that the radar system is able to send and receive signals normally in the downhole environment, and the quality and integrity of the signals are guaranteed. This provides a reliable database for subsequent geological information detection and analysis.

### 3.2. Data Preprocessing

In practical applications, oil wells are usually located in a complex underground geological environment. Substances such as water, oil, gas, and other materials in a formation, as well as fissures, will reflect and refract radar signals, leading to the complication of the signal propagation path. The inhomogeneity of the formation and the characteristics of the multiphase medium further cause signal attenuation and scattering, resulting in signal distortion. For example, in different types of strata, such as sandstone, shale, and carbonate rock, radar signals have different propagation speeds and attenuation characteristics, leading to phase interference and multipath effects, which increase the noise level. As the downhole pressure changes, parameters such as the density and porosity of the medium also change. These changes not only affect the propagation characteristics and reflection strength of the radar signal, they also further exacerbate the presence of noise.

The raw data collected often contain a large amount of environmental noise and various interference signals. These noises and interferences significantly degrade the quality of data, which in turn affects the accuracy of subsequent analysis and processing. Therefore, data preprocessing becomes a key step to improve data quality and signal-to-noise ratios (SNRs). Data preprocessing usually includes noise removal, gain compensation, and baseline correction. The aim is to remove or attenuate unnecessary noise components, correct the amplitude deviation of the signal, and adjust the baseline drift to obtain a purer and more stable signal.

Specifically, denoising is used to clean up the signal by filtering out high-frequency noise. Gain compensation, on the other hand, is used to correct signal amplitude distortion due to sensors or transmission paths. Baseline correction, on the other hand, is used to remove baseline drift from the signal, ensuring that the signal is centered on a zero baseline. These preprocessing steps not only improve the overall quality of the data, but also lay the foundation for subsequent signal analysis and processing, ensuring the accuracy and reliability of the study results.

#### 3.2.1. Denoising

In this study, the weighted moving average (WMA) filtering method was used to denoise the original signals. Weighted moving average filtering is a filtering technique based on a sliding window. By assigning different weights to the data points in the window, the weighted average is calculated, thus realizing signal smoothing and noise suppression. Compared with the simple moving average (SMA) method, weighted moving average filtering can more effectively retain the key features of the signal, reduce the edge effect, and improve the filtering effect.

#### 3.2.2. Rationale

The basic formula for weighted moving average filtering is written as follows (20):

(20) $$Y_{i}=\frac{\sum_{j=i-\frac{N}{2}}^{i+\frac{N}{2}}w_{j}x_{j}}{\sum_{j=i-\frac{N}{2}}^{i+\frac{N}{2}}w_{j}}$$

The choice of weight coefficient, *ω_j_*, directly affects the filtering effect. Common weight assignment methods include linear decreasing weights, Gaussian weights, and so on. In this study, linear decreasing weights were used, i.e., the weight at the center point of the window is the largest and decreases gradually on both sides. This type of weight assignment can better retain the local characteristics of the signal, while effectively suppressing high-frequency noise.

#### 3.2.3. Baseline Drift

Baseline drift is a slow change in the signal baseline caused by temperature changes, power supply fluctuations, and other factors during a long period of signal acquisition. Baseline drift affects the true amplitude of the signal, which in turn affects the analysis results. Baseline correction is designed to eliminate this slowly changing baseline drift and return the signal to a true zero baseline.

**Method of baseline correction** **:**

In this study, a sliding average filtering method was used to estimate the baseline of the signal, and then the baseline correction was realized by subtracting the estimated baseline from the original signal:

(21) $$x_{corrected}(t)=x(t)-\hat{b}(t)$$

where $\hat{b}(t)$ is the baseline estimated by sliding average filtering.

**Figure 28:**
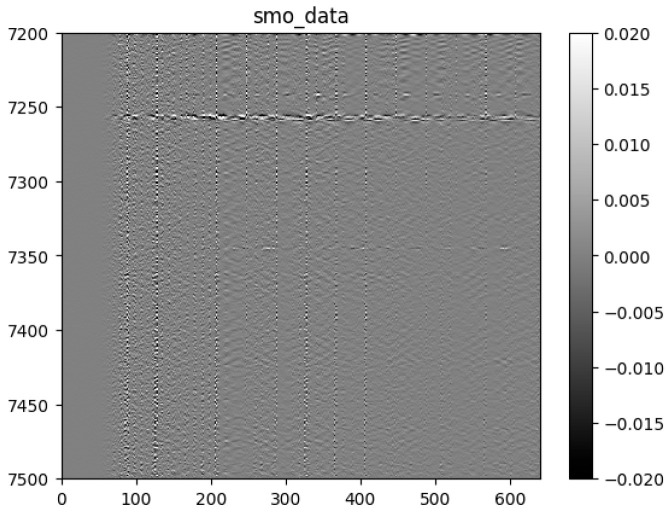
Original data.

The original radar signal image (Figure 28) contains a large amount of high-frequency noise and interference, and overall exhibits a longitudinal, high-contrast streak characteristic of a high noise level. Especially on the time axis, the random interference and background noise in the signal are obvious, resulting in the main features of the signal being submerged or blurred. In the depth direction, the signal waveform is also affected by multipath effects and ambient noise, which manifests itself as signal distortion and indistinguishable structural details.

**Figure 29:**
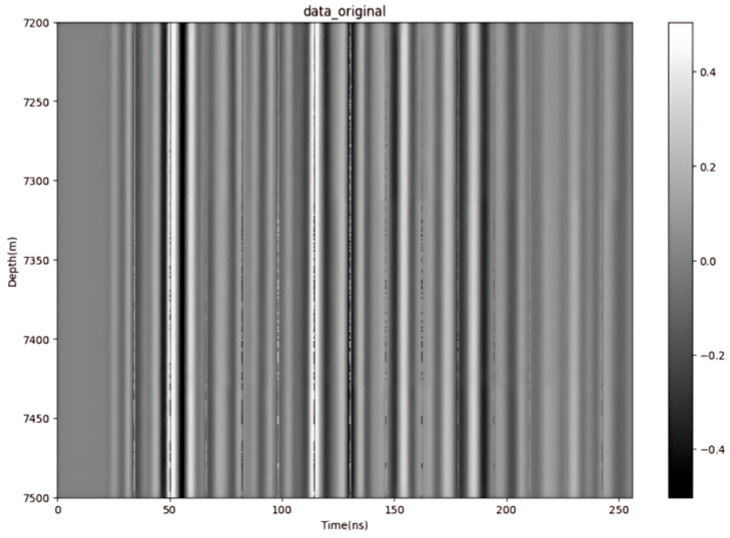
Sliding-window smoothing filter B-scan.

After data preprocessing, the processed radar signal image presents clearer and more regular signal characteristics in Figure 29. The background noise is significantly suppressed, the main structural information and reflection signals in the image are highlighted, and the interference of streaks is weakened. Especially on the time axis and in the depth direction, the continuity and consistency of the signals are significantly enhanced, which helps to identify underground geological features more accurately.

After data preprocessing, the high-frequency noise and random interference in the radar signal are effectively removed, resulting in a significant improvement in the signal-to-noise ratio of the signal and an enhanced ability to analyze the reflection characteristics of the target. This improvement provides a more reliable basis for the subsequent analysis of the geological structure.

## 4. Verification of Experimental Results

### 4.1. Data Validation

The collected radar data were compared and analyzed with the geological drilling data provided by Sinopec. The results show that the detection data of the borehole radar system matched well with the third-party data, especially the reflection characteristics at the casing at 7250 m below ground level in the key stratum, which shows high consistency, which verifies the detection accuracy of the system.

**Figure 30:**
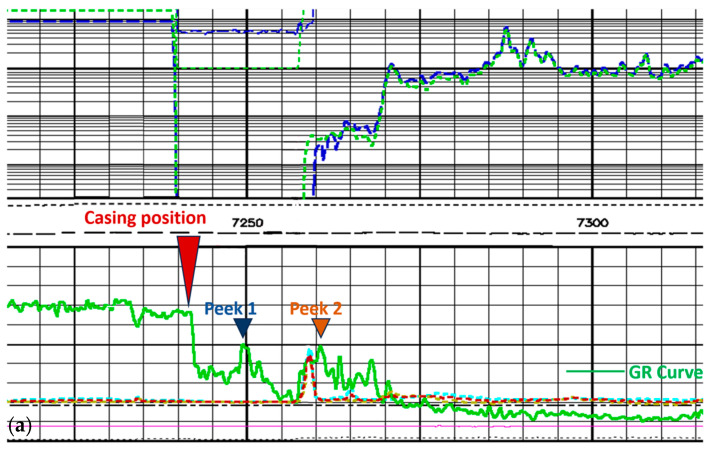

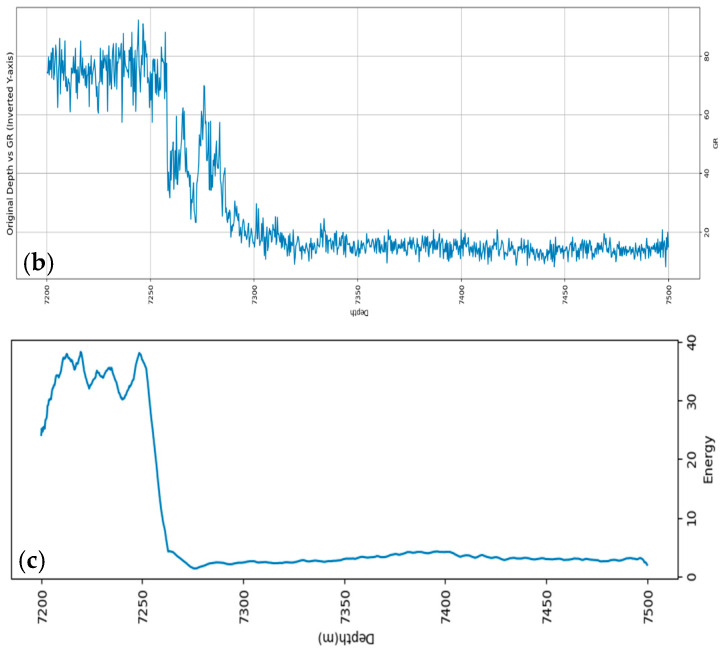
Comparison of third-party data: (**a**) casing comparison in third-party data; (**b**) measured GR data; (**c**) measured energy data.

In Figure 30a, it can be noticed that there is an abrupt change in the GR value at 7240 m. This is due to the fact that after the radar leaves the casing, it enters the sediment mixing zone. This leads to an abrupt change in the GR value. Meanwhile, the corresponding abrupt change in the GR value can also be found at 7256 m in Figure 30b,c. And the length of the radar itself in the well is up to 16 m, so the measured data and the third-party data have an error of about 15 m in depth. In Figure 30a, regarding the third-party data, two small peaks of the GR value can be found after leaving the casing. The measured GR curve (Figure 30b) also corresponds perfectly. The depths of the peaks are 7260 m and 7283 m. The depths of the third-party data are 7250 m and 7261 m. Table 2 shows the errors. The two small peaks occurred because of the small amount of target material mixed in the sediment.

The results show that the detection data of the borehole radar system are in high agreement with the third-party data. The correlation of the calculated GR curve reaches 0.92. The reflection characteristics at the casing at 7250 m below ground especially show a high degree of consistency. And the depth error of the key bottom information is 0.013%. This further verifies the reliability of the radar system in the complex environment of 7500 m underground. It shows that the radar system can not only operate stably but also accurately reflect the underground structural characteristics. This provides strong support for high-precision geological exploration.

### 4.2. System Performance Evaluation

The improved borehole radar system shows significant improvements in terms of depth detection capability, anti-interference performance, and data accuracy. Compared with the traditional system, the improved system is able to capture the detailed features of deep geological structures more clearly, which helps to improve the efficiency of detection of oil and gas resources.

## 5. Conclusions

The harsh environments of deep wells greatly affect the transmission of signals. In this paper, borehole radar is successfully applied for the first time to geological exploration at a depth of 7500 m in the Shunbei area. And compared with the third-party data, the error rate is controlled at about 0.01%, and the correlation of the GR curve is as high as 0.92. We use FPGA as the core, which also has the advantages of easy implementation and low cost. The experimental results show that the borehole radar has higher signal penetration and data accuracy in deep exploration, which verifies the reliability of the system. Future research work will focus on further optimizing the signal processing algorithm and expanding the application range of the system to adapt to more complex geological conditions.

Compared with other geophysical systems, in the radar system presented in this paper, the radar signal sampling mode and the communication mode between the well and the ground are reasonably designed, maximizing the use of the space in the protective sleeve. This makes it possible to realize high-power and high-pulse communication in the well, thus improving the communication quality of the radar in the well. The radar data finally obtained are highly consistent with third-party data.

## Figures and Tables

**Table 1:** The relationship between the detection depth of a shock-type pulse ground-penetrating radar and the parameters of the emitted source pulse.

Depth	Pulse Height (ns)	Center	Depth Resolution
0–0.25	0.50	2000	0.025
0.25–0.5	1.00	1000	0.05
0.5–1.0	2.00	500	0.1
1.0–2.0	4.00	250	0.2
2.0–4.0	8.00	125	0.4
4.0–8.0	16.00	63	0.8

**Table 2:** Errors in third-party data comparisons.

Characteristic Point	Third-Party Data (m)	Measured Data (m)	Length After Radar	Error
0–0.25	7240	7257	7241	0.013%
0.25–0.5	7250	7265	7249	0.013%
0.5–1.0	7261	7285	7259	0.025%

## Data Availability

Data are contained within the article.
